# Feeding behavior innovation increases foraging efficiency in the Amur falcon but may be a threat to Asian particolored bats

**DOI:** 10.1002/ece3.9272

**Published:** 2022-09-11

**Authors:** Lei Feng, Jingjing Li, Hexuan Qin, Yingying Liu, Hui Wu, Jiang Feng, Tinglei Jiang

**Affiliations:** ^1^ Jilin Provincial Key Laboratory of Animal Resource Conservation and Utilization Northeast Normal University Changchun China; ^2^ Key Laboratory of Vegetation Ecology of Education Ministry, Institute of Grassland Science Northeast Normal University Changchun China; ^3^ College of Life Science Jilin Agricultural University Changchun China

**Keywords:** bats, behavioral innovations, birds, energy intake, foraging efficiency

## Abstract

Behavioral innovations are rare and infrequent in the natural world, but they are pivotal for animals to respond to environmental changes. The ecological benefits of these innovations remain unknown, especially in wild populations. Here, two foraging strategies and three eating behaviors of the Amur falcon (*Falco amurensis*) were observed during predation on Asian particolored bats (*Vespertilio sinensis*) across 3 years. We demonstrated that an eating behavioral innovation in *F. amurensis* increased the foraging efficiency of *V. sinensis* more than twofold during 3 consecutive years. This showed that changes in feeding behavior by a bird strongly influenced the rate of energy intake. Since predation on bats by falcons mainly occurred during the lactation and post‐lactation of bats, this may have a certain level of negative effect on the bat population.

## Graphical abstract

In this study, two forage strategies and three eating behaviours in the Amur falcon (*Falco amurensis*) were observed during predation on Asian particoloured bats (*Vespertilio sinensis*) across three years. Moreover, we demonstrated that an eating behaviour innovation in *F*. *amurensis* increased foraging efficiency on *V*. *sinensis* more than twofold during three consecutive years. However, behaviour innovations in Amur falcon may suppress bat population size.

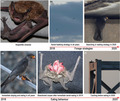

## 1 INTRODUCTION

Behavioral innovations play a critical role in how animals cope with environmental changes, and such innovations may ultimately affect their fitness (Mazza & Guenther, 2021). Since birds have relatively high cognitive abilities, feeding innovations are common in birds, especially in Passerines (Griffin & Guez, 2014). However, understanding the functions of such innovations remains a challenge, as this may require long‐term field observation or alternative experimental assays in the laboratory (Griffin & Guez, 2014; Lefebvre et al., 2016). Moreover, with the exception of Passerines, little is known about the processes, driving forces, and adaptive value of feeding innovations in the wild (Griffin & Guez, 2014).

Raptors are the most common diurnal predators of bats despite bats having long been considered to lack natural predators (Mikula et al., 2016). However, except for a few predators that specialize in hunting bats, such as *Macheiramphus alcinus* and *Falco rufigularis*, almost all avian predators of bats are opportunistic (Ferguson‐Lees & Christie, 2010; Mikula et al., 2016). Bats are difficult to observe because they are highly mobile and nocturnal. Bats account for about one‐fifth of the species diversity of mammals. Although there are many anecdotal reports of birds preying on bats (Mikula et al., 2016), there are few detailed studies concerning this behavior (Black et al., 1979; Brighton et al., 2021; Fenton et al., 1994; Lee & Kuo, 2001; Roberts et al., 1997). Thus, little is known concerning how birds change or innovate their feeding behavior to predate upon bats. Moreover, previous studies have indicated that bat behavior and population dynamics are influenced relatively little by avian predators, especially in temperate zones (Lee & Kuo, 2001; Rodriguez‐Durán & Lewis, 1985), but such as a view is debatable. The accumulated evidence suggests that predators may have impacted the behavior and the evolution of nocturnality in bats, especially in tropical zones (Arndt et al., 2018; Lima & O'Keefe, 2013; Mikula et al., 2016), but their effects on the population sizes of bats are still unknown.

**FIGURE 1:**
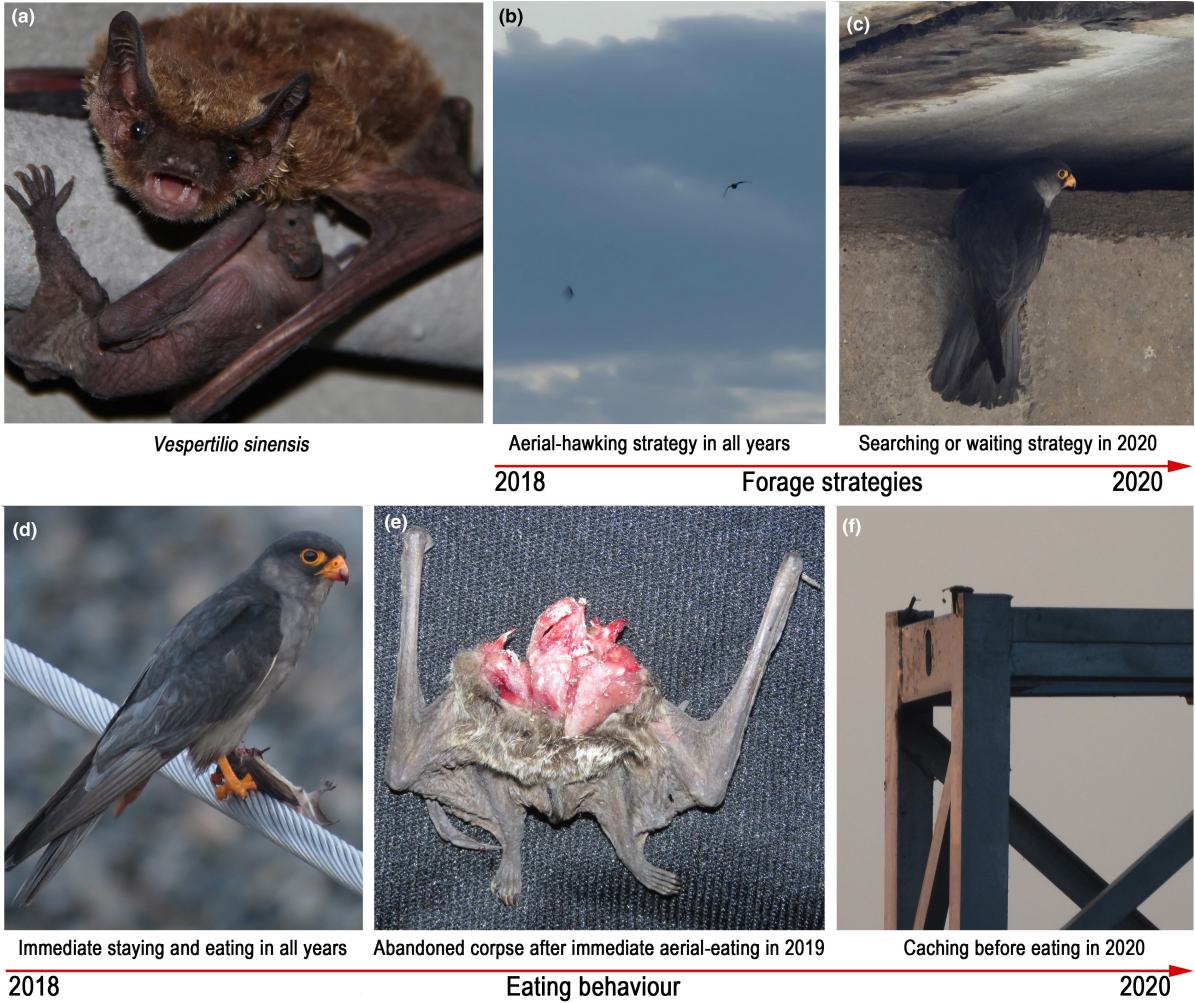
(a) A mother and juvenile of *Vespertilio sinensis*. (b) A falcon (top) hunting a bat (bottom) using the aerial‐hawking strategy at dusk; this strategy was present in all years. (c) A falcon hunting a juvenile using searching or waiting strategy in the daytime in 2020. (d) A falcon eating a bat after foraging. (e) An abandoned corpse of a bat after immediate aerial eating by a falcon in 2019. (f) Two bats were cached before being eaten by falcons in 2020.

On 10 August, 2018, we first observed that the Amur falcon (*Falco amurensis*) regularly preyed on a maternal colony of Asian particolored bats (*Vespertilio sinensis*) under an overpass in Acheng district, Harbin city, northeast China (Figure 1a). This provided a unique opportunity to study the relationship between a bird predator and a bat prey in a wild population. The aims of this study were to: (1) describe the feeding behavior of *F. amurensis* and the anti‐predator behavior of *V. sinensis*; (2) investigate the effects of feeding behavior innovations on the foraging efficiency of *F. amurensis*; and (3) assess the effects of predation of *F. amurensis* on the population size of *V. sinensis*.

## 2 MATERIALS AND METHODS

### 2.1 Study sites and subjects

The study was carried out in a nursery colony of Asian particolored bat (*V. sinensis*) that roosted in gaps of a highway bridge (127°32′E, 45°32′N) in Acheng, Heilongjiang Province, Northeastern China (Figure S1). *V. sinensis* migrates to the bridge in late June and leaves in early September every year and has stayed in this location for at least 10 years. We studied the population for 6 years and observed that *F. amurensis* has regularly preyed on *V. sinensis* since 10 August, 2018.

### 2.2 Foraging observations

The study was carried out from 14 August to 3 September, 2018, and from 10 July to 18 August in both 2019 and 2020. The experimental periods in 2018 were inconsistent with those in both 2019 and 2020 because *F. amurensis* started to prey on *V. sinensis* during mid‐August in 2018. Foraging observation was conducted each day but was canceled when there was rain at dusk or in the daytime.

Normally, we arrived at the bridge 1 h before sunset and began observing the foraging behavior of falcons after the first bat emerged. We stopped observing when the emergence of bats was over at dusk, and we recorded the duration of observation. One experimenter stood at an ideal position on the bridge in order to see clearly the area where falcons preyed on bats. Another experimenter recorded hunting and eating behaviors by a video camera (COOLPIX P1000, Nikon Corp). The average number of falcons was approximately three per day at dusk, and we could not distinguish individuals. During each day at dusk, all falcons arrived at the bridge before bats emerged, and no falcons left during the observation period. In this case, we counted the total number of bats preyed on by falcons for calculation of foraging efficiency.

During lactation and part of post‐lactation of bats in 2019 and 2020, we found that *F*. *amurensis* preyed on the juvenile bats leaving the crevices of the daily roost. It was unclear why these bats crawled out from the crevices of their roosts. Unlike adult bats, these juvenile bats were flightless and could only crawl on the piers of the overpass. Thus, they were vulnerable to predators. Normally, we arrived at the bridge to observe *F*. *amurensis* preying on juvenile bats around sunrise. If there were no falcons for an hour, we finished our observations and restarted at 13:00 p.m. until an hour before sunset. At 1 h before sunset, we arrived on the bridge and started to observe the foraging behavior of falcons after the first bat emerged.

Based on previous studies of this bat population (Yin, 2020), we divided the observation period into lactation and post‐lactation periods. The period from July 1 to July 31 in 2019 and 2020 was defined as the lactation period of the bats, while the remaining observation period was defined as the post‐lactation period of the bats. At dusk during each observation period, we recorded the time of the first appearance of *F*. *amurensis*. We also recorded the first attack time and the last capture time of the falcons. We defined the difference between the two times as the daily predation duration of *F*. *amurensis*. We calculated the capture success rate of the falcons using the number of hunted bats divided by the total number of attacks. We also calculated the eating duration of *F*. *amurensis* on *V*. *sinensis* and defined it as handling time.

In this study, we observed two foraging strategies in *F. amurensis*: aerial hunting at dusk (Figure 1b; Video S1), and the searching or waiting strategy for predation on juveniles (pups or newly volant bats) in the daytime (Figure 1c; Video S2). Aerial hunting was observed in all years, but the searching or waiting strategy was only observed in 2020. The searching or waiting strategy is described briefly as follows. During the daytime (not dusk or dawn), *F*. *amurensis* perched on a wire, an iron communications shelf near the roost of the bats, or the wires under the overpass. The falcons waited and searched for juvenile bats under the overpasses. If falcons found a bat outside the roost, the falcons would prey on the juvenile bats (Video S2). We did not observe distinct anti‐predator behavior in these juvenile bats, but we observed that some juvenile bats tried to climb back to the roost. During post‐lactation, we also found that when some newly volant bats flew out of the roost they would be preyed on by falcons waiting outside the roost. Most of the newly volant bats were captured by falcons during the daytime despite the bats performing anti‐predation behaviors such as changing the direction of flight, albeit with weak volant skills. If a newly volant bat successfully flew back into the crevice under the overpass, the falcons would search the crevice or wait for the next bat (Figure 1c; Table 1).

**TABLE 1:** Descriptions of foraging strategies and eating behaviors of *Falco amurensis*

Forage strategy or eating behavior	Description
Aerial hunting	Falcons hunt bats during flight.
Searching or waiting strategy	Falcons perched on wires or other artificial structures to search and wait for juvenile bats during the daytime and catch them as the bats crawled out or flew out from crevices of the roost.
Perching eating	Falcons immediately stopped hunting to consume the captured bat.
Aerial eating	Falcons immediately consumed the captured bat during flight.
Caching	Falcons immediately killed and cached the captured bats on the artificial towers, then continued to hunt.

We also observed three eating behaviors after hunting: perched eating (Figure 1d; Video S3), aerial eating (Figure 1e), and caching captured bats (Figure 1f; Video S4). Perched eating was the most common in all years, while aerial eating and caching captured bats were observed only in 2019 and 2020, respectively. In 2019, we found that *F*. *amurensis* cached captured bats after successful predation, but this behavior was only recorded twice on video. In 2020, we found *F*. *amurensis* again using this particular behavior to deal with captured bats. It is difficult to accurately distinguish between individual falcons, but we are sure that more than one falcon used caching behavior.

### 2.3 Estimation of population size for bats

The bridge includes 14 archways. Here, “archway” was defined as the region surrounded by two beams (Figure S1). Every archway contains 12 crevices where bats roost. The crevices are about 5 cm deep, and bats normally roost one by one along the crevice (Figure S1). In this case, we could survey population size by direct counting. During the daytime on 15 August, 2020, we stood on scaffolds to count the bats using a flashlight. In order to reduce human interference with the bats, we only surveyed three archways. Moreover, it was difficult to set up the scaffold stably under some archways. The average bat number was 450 per archway, indicating a population size of about 6300 individuals.

### 2.4 Calculation of foraging efficiency

Since individuals of *F. amurensis* could not be distinguished, foraging efficiency was calculated using the number of bats divided by the number of falcons and by the duration of observation. The efficiency was thus expressed as the number of bats that each falcon preyed on per unit time (hour) in 2019 and 2020. Successful predation was observed nine times at dusk in 2018. The sample size was very small and thus was not suitable for performing time‐series analysis. Thus, foraging efficiency in 2018 was not analyzed or displayed.

### 2.5 Observed and predicted numbers of bats captured by falcons

We obtained the average number of bats captured per day at dusk based on the observation data in 2018, 2019, and 2020. Bats were captured by falcons from 20 June to 30 August (72 days) in 2019 and 2020. Thus, we predicted the total number of bats killed by falcons using the average number of bats captured per day at dusk multiplied by 72 days in 2019 and 2020. Because *F. amurensis* were observed preying on *V. sinensis* since 10 August, 2018, the duration of predation of falcons was 21 days (from 10 August to 30 August).

### 2.6 Statistical analyses

In order to test whether a feeding behavior innovation influenced the foraging efficiency of *F. amurensis* at dusk, time‐series analysis was performed in R 4.0.3 (Team, 2020) based on the framework presented by Wauchope et al. (2021). Here, we did not use data from 2018 because we only made nine observations at dusk in that year. Time‐series data from 2019 and 2020 were analyzed separately. Missing values were imported using the na.approx function in the package “zoo” (Zeileis & Grothendieck, 2005). Mann–Kendall trend tests were performed using the Mann–Kendall function in the package “Kendall” (McLeod, 2011). Sen's slope was calculated by the sens.slope function in the package “trend” (Pohlert, 2020). We also identified a point at which the values in the data changed using the Pettitt. test function in the package “trend” (Pohlert, 2020). Additionally, we employed the ptestg function with the robust g test in the package “ptest” to estimate the periodicities of time‐series data from 2019 and 2020 separately (Lai & Mcleod, 2016).

We also performed an intervention analysis to determine whether the appearance of caching behaviour affected foraging efficiency at dusk. Stationarity was estimated using an autocorrelogram. The randomness for time‐series data from 2020 was tested using the Box.test functions in the package “aTSA” (Qiu, 2015). We used the auto.arima function in the package “forecast” to find the appropriate ARIMA model (Hyndman & Khandakar, 2008). In the model, foraging efficiency at dusk was used as the dependent variable, and caching behaviour appearance was defined as the intervention variable. Values of the intervention variable were presented as 0 or 1. We found that an ARIMA (0,0,1) model was the most appropriate for the time‐series data from 2020. After model fitting, we determined whether the fitted model was validated using the tsdiag function in the package “forecast” (Hyndman & Khandakar, 2008). Finally, we calculated the degree of change in foraging efficiency due to the appearance of caching behavior based on intervention coefficients from the models.

## 3 RESULTS

### 3.1 Foraging behavior of *F. amurensis* and the anti‐predator behavior of *V. sinensis*

At dusk, *F. amurensis* arrived at the overpass 20.76 ± 20.45 min (*n* = 97) prior to sunset. Then, *F. amurensis* perched on a wire beside the overpass to wait for the emergence of *V. sinensis* and used an aerial hunting strategy (Figure 1b) to hunt bats at dusk. This hunting pattern was observed since 2018. Bats often emerged from the roost before sunset during the lactation period, but this phenomenon was rarely observed during the post‐lactation period. The time of bats' emergence gradually became delayed from lactation to post‐lactation. The initial stage of the emergence consisted of bats emerging sporadically from the daily roost at intervals of more than 1 min; the longest interval was more than 10 min between two emerging bats. We did not observe that the emerged bats formed close columns because of the small population size (Video S1 and S4). *F. amurensis* began to hunt after the first bat flew out from roost. In order to capture *V. sinensis*, *F. amurensis* persistently chased *V. sinensis* and changed direction with the bat. In many successful cases, *F. amurensis* flew upward as normal, then dived suddenly and accelerated to attack the focal bat. In the beginning, when only a few bats were flying out from the roost, a bat may have been attacked by several falcons, or a single bat may have been attacked several times by a single falcon. The maximum record was that of a bat that was chased seven times by a falcon in 1 min. We did not observe any of the bats that first emerged to be successfully captured by a falcon. We recorded 133 successful predation events of *F. amurensis* from 2018 to 2020. The number of attacks ranged from 1 to 20. The total number of attacks was 459, and thus the capture success rate was 29%. For the bats, we also observed an apparent presence of escape maneuvers (i.e., aerial dodges and precipitous drops) in *V. sinensis* to avoid *F. amurensis*. Additionally, *V. sinensis* also produced distress calls when they were caught by *F. amurensis*.

The predation duration of *F. amurensis* ranged from 3 to 37 (21.23 ± 9.34) minutes. The predation duration of the falcons gradually shortened from the lactation to the post‐lactation periods of the bats. During perching eating, the head of bats was often eaten first, and falcons would usually eat all of the bat's body except the wing membrane, although sometimes they would also eat the wing membrane. Moreover, the average eating time of *F. amurensis* after catching a bat was 8.95 ± 2.76 min (*N* = 19).

### 3.2 Effects of feeding behavior innovations on the foraging efficiency of *F. amurensis*

**FIGURE 2:**
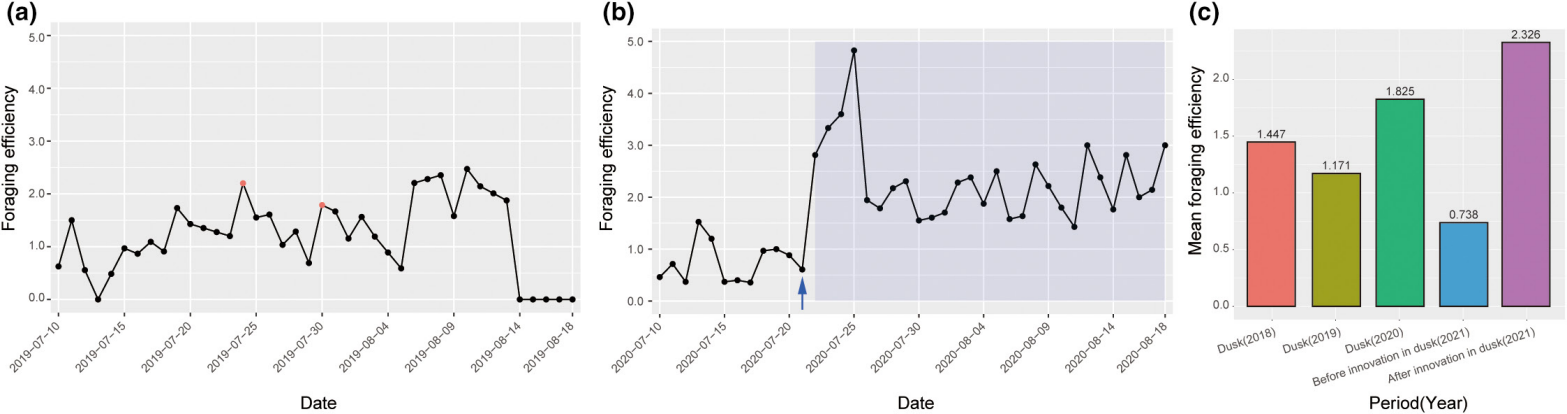
(a) Foraging efficiency of falcons across days from 10 July to 18 August in 2019. The two red points represent the appearance of immediate aerial‐eating behavior after foraging. (b) Foraging efficiency of falcons across days from 10 July to 18 August in 2020. The light blue area represents the appearance of caching before eating behavior after hunting. The blue arrow represents a significant change point identified by the Pettitt test. (c) The mean foraging efficiency of falcons in different periods in different years.

There was no periodicity in the time‐series data from 2019 (robust g test: *p* = .054) or 2020 (robust g test: *p* = .292) separately, but an ascending trend of foraging efficiency in *F. amurensis* was detected in 2020 (Mann–Kendall trend test: *τ* = 0.405, *p* = .0002) rather than in 2019 (Mann–Kendall trend test: *τ* = 0.118, *p* = .293). Moreover, significant change points were detected in foraging efficiency in 2020 (Pettitt test: U* = 334, *p* < .0001; 21 July, 2020; Figure 2b) rather than in 2019 (Pettitt test: U* = 170, *p* = .142; Figure 2a), suggesting a significant change in foraging efficiency after 21 July, 2020. The change point identified by the Pettitt test was consistent with the appearance of caching captured bats (from 22 July to 18 August, 2020, the light blue area in Figure 2b).

Time series in foraging efficiency were stationary based on the autocorrelograms. The time series of foraging efficiency were not purely random sequences in 2020 (*X* ^2^ = 27.328, *df* = 4, *p* < .0001). After model fitting for the time series of foraging efficiency in 2020, the fitted model was denoted as Model (1):

(1) Yt=0.715+1.624Xt+εt+0.344εt−1

ε*t ~* NID (0, 0.407).

Here, *Yt* is the foraging efficiency and *Xt* is the intervention variable (caching behaviour appearance or absence). The fitted model for the time series in 2020 was valid (all *p* > .05). The mean foraging efficiency before the innovation in 2020 was 0.738 (Figure 2c), and the intervention coefficient from Model 1 was 1.624. These results showed that the appearance of caching behavior led to a 2.2‐fold increase in foraging efficiency (Figure 2c).

### 3.3 Effects of predation of *F. amurensis* on the population size of *V. sinensis*

**FIGURE 3:**
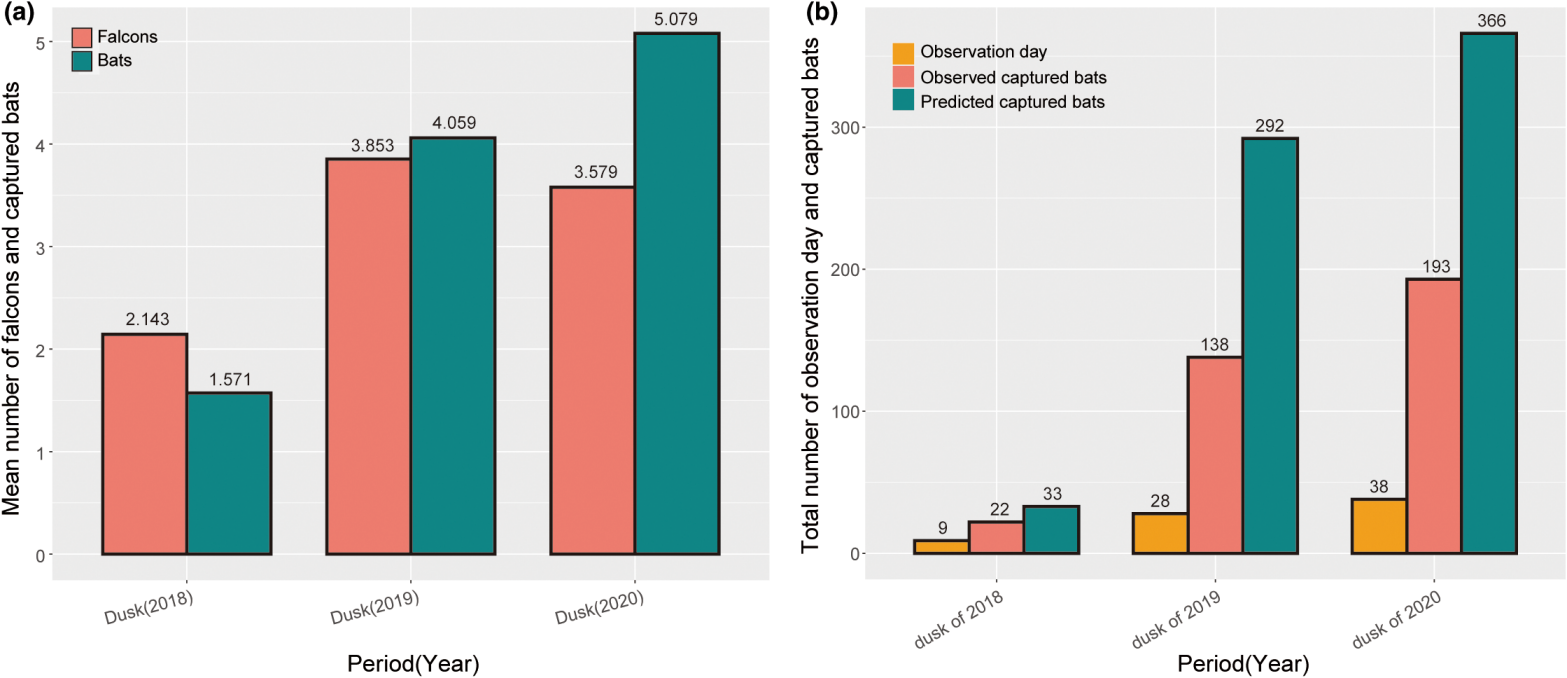
(a) The mean number of falcons appearing to forage and captured bats per day at dusk in different periods in different years. (b) The total number of days of successful predation was observed, and bats captured by falcons based on observations and predictions in different periods in different years.

The average numbers of falcons per day at dusk were 2.14 ± 0.77, 3.85 ± 1.67, and 3.58 ± 1.57 in 2018, 2019, and 2020, respectively (Figure 3a). The average numbers of bats per day at dusk captured by falcons were 1.57 ± 1.65, 4.06 ± 3.06, and 5.08 ± 3.38 in 2018, 2019, and 2020, respectively (Figure 3a). The observed numbers of bats captured by falcons at dusk were 22, 138, and 193 in 2018, 2019, and 2020, respectively, and the predicted numbers of bats captured by falcons at dusk were 33, 292, and 366 in 2018, 2019, and 2020, respectively. Increasing trends were observed in both observed and predicted numbers of bats across the years (Figure 3b). During the daytime, after a successful hunt, some *F. amurensis* would feed on the captured bats and then re‐search for juvenile bats under the overpass. Sometimes *F. amurensis* would take away the captured bats and return later to hunt again. In the daytime in 2020, the number of juvenile bats captured by *F*. *amurensis* was 252.

## 4 DISCUSSION

In this study, we found that *F. amurensis* regularly preyed on *V. sinensis*. To our knowledge, *F. amurensis* primarily feeds on insects, small amphibians, birds, and small mammals (Pietersen & Symes, 2010). Thus, this was the first record of *F. amurensis* regularly preying on bats, implying that predation on bats by raptors was opportunistic in our specific environment. We found that almost all successful predation events occurred during the departure clustering of *V*. *sinensis*. Departure clustering apparently would dilute the predation risk to individuals of a bat colony (Santos et al., 2016). However, it may be more efficient for predators to hunt bats in a dense group (Brighton et al., 2021). Additionally, the bats that first emerged from the roost maybe those with more agile flight (Thomas & Jacobs, 2013). Therefore, the greater availability of bats may result in the majority of successful predation events occurring during the clustered emergence. The catch success of *F*. *amurensis* that we observed was comparable to those of other diurnal predators of Falconidae, but lower than that in *Falco subbuteo* (Brighton et al., 2021). The variation in catch success is influenced by local conditions; in the *F. subbuteo* example, the extreme prey abundance (about 10 million) and earlier and longer emergence as well as the presence of newly volant *Tadarida brasiliensis* may have resulted in a higher capture success for *F*. *subbuteo* than for other falcons (Lee & Kuo, 2001). In our study, the population of bats was about 6300, and during post‐lactation, although we did find newly volant bats being preyed on by *F*. *amurensis* at dusk, the delayed emergence of *V*. *sinensis* may have resulted in lower capture success for *F*. *amurensis*. Additionally, although *V. sinensis* produced distress calls when they were caught by *F. amurensis*, mobbing behaviors of bats and falcons being startled were not observed from 2018 to 2020. Thus, in addition to the escape behavior of bats in response to pursuit by birds, future quantitative studies should determine how bats use anti‐predator behavior to counter attempted predation by birds.

Here, *V*. *sinensis* often emerged from the roost before sunset during the lactation period (in July), which was earlier than during the post‐lactation period (after July). Our previous study found that more than 65% of the first emergence events of *V*. *sinensis* occurred before sunset during lactation, whereas only about 7% of the first emergence events occurred before sunset during post‐lactation (Feng et al., 2022). The energetic cost of an adult female bat is increased considerably, peaking during lactation (Kunz, 1987). Therefore, the onset of emergence occurred earlier during lactation (Acharya et al., 2015; Arndt et al., 2018). The earlier emergence of bats may increase foraging time in order to maximize food availability, but this would expose the bats to higher predation risk. During the lactation period, the daily predation duration of *F*. *amurensis* was significantly longer than that in the post‐lactation period. Therefore, it may be that the earlier onset of emergence during lactation provided more predation opportunities for diurnal predators.

Behavioral innovation is defined as a solution to a novel problem, a novel solution to an old problem, or exploitation of a food resource not previously part of the diet (Kummer & Goodall, 1985). Our results indicated that a behavioral innovation (i.e., caching captured bats) in *F. amurensis* increased foraging efficiency at dusk more than twofold compared to using the perched eating and aerial eating strategies. These results confirmed that *F. amurensis* can innovate its feeding behavior via learning to maximize the fitness benefits, and this can explain the changes in feeding behavior in this species. Darwinian fitness is fundamentally determined by the rate of energy intake, and therefore should be under intense selection (Boag & Grant, 1981). In this study, the duration of emergence by bats at dusk was very short, with an average of 42.78 ± 13.55 min (*N* = 91). Moreover, the average eating time of *F*. *amurensis* after catching a bat was 8.95 ± 2.76 min (*N* = 19). In this case, perched eating after foraging for *F*. *amurensis* was not optimal because it wasted foraging time at dusk. In 2019, aerial eating after foraging was performed by *F*. *amurensis* at dusk to consume bats more quickly during flight. However, aerial eating was also suboptimal because it not only increased the difficulty of eating but also wasted food, as most of the body of the bat was abandoned (Figure 1e). Alternatively, *F*. *amurensis* also may simply choose to eat only the most nutritious part of the bats (i.e., the heart and brain; Figure 1e). A previous study showed that the head of prey is often eaten first by captive raptors, possibly due to the high‐fat content of the brain (Slagsvold et al., 2010). Moreover, raptors with large gapes could feed on bats rapidly by swallowing the bats whole, and aerial eating is often favored by raptors (Fenton et al., 1994). In our study, *F*. *amurensis* did not swallow the bats whole, and the capture success of *F*. *amurensis* was only 29%. Thus, aerial eating behavior only occurred twice and has not been retained, possibly due to the difficulty of eating, low feed efficiency, and low capture success. In 2020, caching behavior increased both foraging time and food intake because *F. amurensis* had enough time to eat the entire bat body. The increases in energy acquisition achieved with this foraging behavior innovation should be helpful for the survival and reproductive success of *F. amurensis* since they were in the breeding season. Relative to perched eating and aerial eating, caching captured bats after foraging was more economical and thus should be favored by natural selection. Our results also confirmed that caching behavior was used by more than one individual on each observation day at dusk after 22 July, 2020. Although we cannot rule out that this may be an idiosyncratic behavioral trait of particular individuals, here it seems unlikely. If caching behavior was an idiosyncratic behavioral trait of particular individuals, it would have occurred for 3 consecutive years. However, caching behavior in *F. amurensis* was only observed in 2020. Additionally, we believe that caching behavior was an innovation rather than the arrival of a new falcon with caching behavior from elsewhere in 2020. This was because we observed more than one falcon displaying this behavior on a given day despite the fact that it did not happen very often. Moreover, we observed the caching behavior every day in 2020. Thus, it seems highly unlikely to observe caching behavior every day if there was only a single falcon using the behavior. In conclusion, these results confirmed that caching behavior in *F. amurensis* may have been acquired from learning and experience gained during 3 consecutive years, and thus functioned to optimize the rate of energy intake.

In addition to the foraging behavior innovation, *F. amurensis* also hunted juvenile bats using novel foraging strategies during the daytime in 2020, a behavior that saves energy during foraging and increases the available foraging time for *F. amurensis* for the following reasons. Relative to dusk, daytime is long enough for *F. amurensis*. Moreover, juvenile bats are much easier to hunt than adults. Finally, predation on pups or newly volant bats may be energy‐saving relative to the aerial hunting strategy because *F. amurensis* waited for lone bats away from crevices and normally only performed a flight to capture the bats. The results were consistent with the predictions of optimal foraging theory (OFT) stating that predators should favor hunting juvenile, old, and sickly prey to minimize the energy costs of foraging (Pyke et al., 1977). The juvenile bats were also likely to die even if they were not preyed upon by falcons because most of them rarely went back to the crevices of their roost. Hence, predation on these juvenile bats by the falcons may not have had negative effects on the bat population, but it may have helped to fulfill the food requirements for the predators.

Bats can be captured by many taxa, including fish, amphibians, reptiles, birds, and mammals, but most predation on bats has been observed in owls and other avian predators, and such predation may be opportunistic (Lima & O'Keefe, 2013). Previous studies have shown that most predation on bats by avian predators has accounted for <2% of the total colony (Lee & Kuo, 2001; Rodriguez‐Durán & Lewis, 1985). However, more than 90% of bats taken by avian predators are killed by owls, and only 5% fall prey to diurnal raptors (Speakman, 1991). Here, since the appearance of caching behavior, about 366 adult bats would have been captured at dusk in 2020 (Figure 3c), accounting for about 6% of the total colony. Moreover, at least 252 juvenile or newly volant bats were preyed on by *F. amurensis* during the daytime in 2020. Thus, the results implied that the effects on bat populations of opportunistic predators with innovative hunting strategies may be significant.

## 5 CONCLUSION

In summary, our study demonstrated that feeding behavior innovations in birds can increase foraging efficiency, but the innovation may only slightly impact the population sizes of bats. Therefore, our study provides evidence for fitness increases due to behavioral innovations in a wild bird population. Our results also confirmed that opportunistic predation pressure may reduce the population size of bats. Our observations on predation on bats by birds raise several issues to be explored in an ecological framework. Is predation on bats by birds during the daytime widespread? Moreover, have the behavioral innovations spread throughout the falcon population, and are they transmitted via learning in the context of predation on bats? Additionally, little is known about anti‐predator behavior of bats, and thus the relationship between birds and bats should be clarified. Along with long‐term studies investigating the dynamic behavioral changes in avian predation and anti‐predator behavior of bats, we can then open avenues on the potential relationships between bat prey and avian predators.

## AUTHOR CONTRIBUTIONS

**Lei Feng:** Conceptualization (equal); investigation (equal); methodology (equal); writing – original draft (supporting). **Jingjing Li:** Investigation (equal). **Hexuan Qin:** Investigation (equal). **Yingying Liu:** Investigation (equal). **Hui Wu:** Formal analysis (equal); methodology (equal). **Jiang Feng:** Conceptualization (equal); funding acquisition (equal); supervision (equal). **Tinglei Jiang:** Conceptualization (equal); formal analysis (equal); methodology (equal); supervision (equal); writing – review and editing (equal).

## CONFLICT OF INTEREST

The authors declare no conflicts of interest.

## Supporting information


**Appendix S1** Supporting Information

Video S1

Video S2

Video S3

Video S4

## Data Availability

Data are available in the Dryad Digital Repository: https://doi.org/10.5061/dryad.2rbnzs7p9.
