# Supplementary material for: Feeding behavior innovation increases foraging efficiency in the Amur falcon but may be a threat to Asian particolored bats
Source: Ecol Evol. 2022 Sep 11;12(9):e9272. doi: 10.1002/ece3.9272 (PMC9465184; doi:10.1002/ece3.9272)
Supplement: Supplementary file 1 — Appendix S1 Supporting Information [file ECE3-12-e9272-s004.docx]

Supplementary Information for

**Feeding behaviour innovation increases foraging efficiency in Amur falcon but may be a threat to Asian particoloured bats**

Lei Feng, Jingjing Li, Hexuan Qin, Yingying Liu, Hui Wu, Jiang Feng and Tinglei Jiang


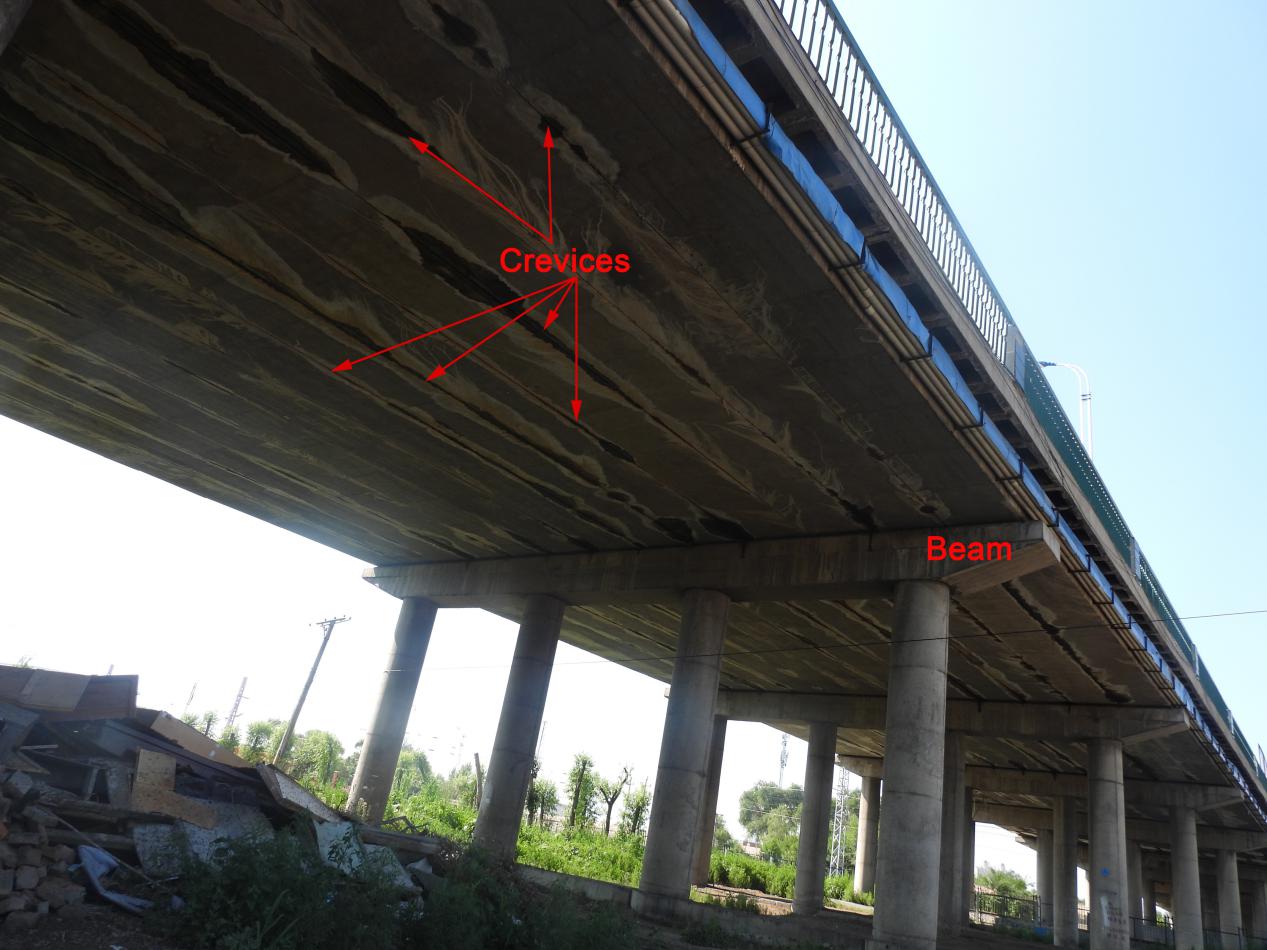


Figure S1. *Vespertilio sinensis* roost in crevices of a highway bridge. Here, “archway” was defined as the region surrounded by two beams, and every archway contains 12 crevices.

**VIDEOS**

**Supplementary Video S1.** *Falco amurensis* hunted *Vespertilio sinensis* using the aerial-hawking strategy and cached the bats before eating to quickly continue hunting.

**Supplementary Video S2.** *Falco amurensis* used a searching or waiting strategy to hunt juvenile in the daytime in 2020.

**Supplementary Video S3.** *Falco amurensis* eating a juvenile bat in the daytime in 2020.

**Supplementary Video S4.** *Falco amurensis* immediately stayed and ate bats after hunting successfully.
